# Improving Power of Genome-Wide Association Studies with Weighted False Discovery Rate Control and Prioritized Subset Analysis

**DOI:** 10.1371/journal.pone.0033716

**Published:** 2012-04-09

**Authors:** Wan-Yu Lin, Wen-Chung Lee

**Affiliations:** 1 Institute of Epidemiology and Preventive Medicine, College of Public Health, National Taiwan University, Taipei, Taiwan; 2 Department of Biostatistics, University of Alabama at Birmingham, Birmingham, Alabama, United States of America; 3 Research Center for Genes, Environment and Human Health, National Taiwan University, Taipei, Taiwan; Johns Hopkins University, United States of America

## Abstract

The issue of large-scale testing has caught much attention with the advent of high-throughput technologies. In genomic studies, researchers are often confronted with a large number of tests. To make simultaneous inference for the many tests, the false discovery rate (FDR) control provides a practical balance between the number of true positives and the number of false positives. However, when few hypotheses are truly non-null, controlling the FDR may not provide additional advantages over controlling the family-wise error rate (e.g., the Bonferroni correction). To facilitate discoveries from a study, weighting tests according to prior information is a promising strategy. A ‘weighted FDR control’ (WEI) and a ‘prioritized subset analysis’ (PSA) have caught much attention. In this work, we compare the two weighting schemes with systematic simulation studies and demonstrate their use with a genome-wide association study (GWAS) on type 1 diabetes provided by the Wellcome Trust Case Control Consortium. The PSA and the WEI both can increase power when the prior is informative. With accurate and precise prioritization, the PSA can especially create substantial power improvements over the commonly-used whole-genome single-step FDR adjustment (i.e., the traditional un-weighted FDR control). When the prior is uninformative (true disease susceptibility regions are not prioritized), the power loss of the PSA and the WEI is almost negligible. However, a caution is that the *overall* FDR of the PSA can be slightly inflated if the prioritization is not accurate and precise. Our study highlights the merits of using information from mounting genetic studies, and provides insights to choose an appropriate weighting scheme to FDR control on GWAS.

## Introduction

The issue of large-scale testing has caught much attention with the advent of high-throughput technologies such as whole genome single-nucleotide polymorphism (SNP) arrays. In genome-wide association studies (GWAS), researchers are often confronted with a large number of SNPs. Two measures are commonly used to quantify the overall error rates when making simultaneous inference for the many SNPs. One is the family-wise error rate (FWER), defined as the probability of committing at least one type-I error from among a family of tests. Methods such as the Bonferroni correction and the Holm's step-down procedure [1] can be used to control the FWER. The other commonly used measure is the false discovery rate (FDR), defined as the expected ratio of the number of false rejections to the number of total rejections [2]–[6]. When the number of true null hypotheses (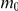) is smaller than the total number of hypotheses (*m*) (i.e., not all the null hypotheses are true), the FDR is smaller than or equal to the FWER [2]. Therefore, given a same nominal control level, controlling the FDR is less stringent than controlling the FWER. Controlling the FDR can provide a more practical balance between the number of true positives and the number of false positives. The FDR controlling has been widely applied to many gene expression data sets, in which the proportions of signal genes (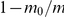) are usually not small.

For some GWAS where the proportions of signal SNPs are extremely small (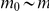) [7], controlling the FDR provides no more benefits than controlling the FWER [8]. Fortunately, appropriately utilizing information from mounting genetic studies can improve this. If researchers have informative prior knowledge, weighting tests according to this prior information can substantially improve the power of a study [8], [9]. There are two approaches to weight tests. One is the ‘weighted FDR control’ (WEI) [10]. The *p* values are weighted directly based on prior knowledge, and then the Benjamini and Hochberg's FDR controlling [2] or the Storey and Tibshirani's FDR controlling [4] is applied to the weighted *p* values. A study has shown a prominent benefit of using prior linkage results to weight the *p* values of association tests [9]. The second approach is the ‘prioritized subset analysis’ (PSA) [11], which has been applied to both GWAS [8] and gene expression data analyses [12]. A researcher first allocates all tests under study into two subsets, based on his/her prior knowledge. A ‘prioritized subset’ comprises tests likely to be the true positives, and a ‘non-prioritized subset’ comprises the remaining tests. The FDR controlling is then applied to the two subsets, respectively.

Appropriately utilizing prior information is crucial to exploring signals, especially when the numbers of tests are going into millions, such as the scenario in GWAS. Both the WEI and the PSA have caught much attention for their advantage to facilitate discoveries from GWAS [8], [9], [11]. However, the comparison between them is not clear. In this work, we make a head-to-head comparison between these two approaches. We compare them with extensive simulations, and demonstrate their use on a real GWAS. Our work can provide insights to choose an appropriate strategy when making simultaneous inference for the many SNPs in GWAS.

## Methods

### Whole-genome Single-step FDR Adjustment (WGA)

The WGA is simply the traditional un-weighted FDR control for GWAS. Suppose that there are a total of 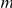 SNPs. Let 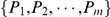 be the set of observed *p* values of the *m* SNPs. To control the FDR at a desired level, say 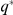, the Benjamini and Hochberg's procedure [2] can be applied to this set of *p* values.

### Prioritized Subset Analysis (PSA)

To perform the PSA and the following WEI, prior information is required to assign each SNP to be ‘more likely a true positive’ or ‘more likely a true negative’. Suppose that we have prior information coming from our biological knowledge, or from findings of data other than that in the current study. Let 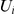 = 1 if the *i*th SNP is located in a chromosomal region supported by prior information and this SNP is thought to be more likely a true positive; 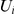 = 0 if this SNP is in a chromosomal region not supported by prior information and it is thought to be more likely a true negative. To perform a PSA, we first allocate all SNPs into two subsets: a ‘prioritized subset’ comprises SNPs likely to be the true positives (*U* = 1), and a ‘on-prioritized subset’ comprises the remaining SNPs (*U* = 0). The observed *p* values of the *m* SNPs are accordingly allocated into two subsets. One comprises the *p* values of the prioritized SNPs, and the other comprises the *p* values of the remaining non-prioritized SNPs. The Benjamini and Hochberg's FDR controlling [2] is then applied to these two subsets of *p* values, respectively.

### Weighted False Discovery Rate Control Procedure (WEI)

There are two weighting schemes for the WEI: ‘binary weighting’ and ‘general weighting’ [10]. The ‘general weighting’ requires a researcher to assign a weight for each and every SNP specifically. To have a parallel comparison between the WEI and the PSA, we here only consider the ‘binary weighting’ for the WEI. In ‘binary weighting’, SNPs thought to be more likely true positives (*U* = 1) are all assigned a same weight (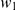), and SNPs thought to be more likely true negatives (*U* = 0) are all assigned another weight (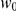).

Let 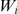 be the weight assigned to the *i*th SNP, *i* = 1, …, *m*. In the binary weighting scheme, 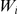 is either 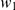 or 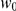. The *p* values are weighted according to 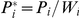, where 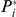 is the weighted *p* value of the *i*th SNP. The Benjamini and Hochberg's FDR controlling [2] is then applied to the set of weighted *p* values 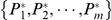. To maintain the FDR at a desired level, the set of weights 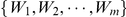 must meet a requirement: 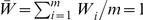 [10]. For the weights in the binary weighting scheme, a researcher can first decide a 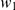 (or 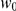), and work out the other one, 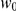 (or 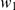), using the constraint 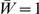. An alternative is to choose a ratio of the two weights, 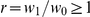, and then obtain 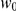 with the constraint:
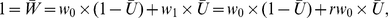
where 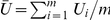. Therefore, 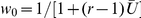 and 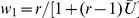.

The choice of *r* reflects the degree of confidence a researcher has toward the prior, which is subjective and is specified by the researcher. (Note that the PSA does not require this parameter, because the PSA simply allocates all SNPs into two subsets without specifying any explicit weight.) If the researcher is confident of the prior information, *r* can be specified larger. If not, *r* should be specified smaller. When *r* = 1, 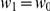, the WEI reduces to the WGA.

### Simulations

We performed simulations to compare the power and the ability to control the FDR of the WGA, the PSA, and the WEI. To provide a practical evaluation on these methods when analyzing GWAS, we followed Li et al. [11] to first simulate GWAS data with similar linkage disequilibrium (LD) patterns as the HapMap data [13], and then analyzed the simulated GWAS data sets with the WGA, the PSA, and the WEI. For the WEI, we followed Genovese et al. [10] to specify *r* at 2, 5, or 10.

#### Simulation program

We used a rapid whole-genome simulation program, the GWAsimulator [14] (http://biostat.mc.vanderbilt.edu/wiki/Main/GWAsimulator), to generate GWAS data sets. The GWAsimulator [14] implements a rapid moving-window algorithm [15] to simulate whole genome case-control or population samples. It faithfully generates SNP genotypes that follow the local LD patterns of the input data. Following Li et al. [11], we used the phased data of HapMap 60 CEU (CEPH samples with ancestry from northern and western Europe) founder subjects as the input data. The total number of SNPs in the input data is 314,174, after merging the Illumina Sentrix HumanHap300 BeadChips (317,503 SNPs) and the HapMap phased data [11], [14].

#### Setting of the disease model

Following Li et al. [11], we let six SNPs be the disease variants. Among the six variants, three have small effects (genotypic relative risk or GRR = 1.34) and the others have relatively large effects (GRR = 1.57). We randomly chose SNPs with minor allele frequencies (MAFs) of 0.25, 0.36, and 0.33 from chromosomes 6, 10, and 5 respectively, as the three small-effect SNPs (Locus 1–3). The three large-effect SNPs (Locus 4–6) with MAFs of 0.43, 0.31, and 0.30 were randomly picked from chromosomes 3, 11, and 4 respectively. This MAF setting mimics the reported risk loci of type 2 diabetes [16], in which the minor alleles were treated as risk alleles. Given the genotypes of the six disease loci, the probability of being affected is 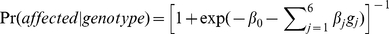, where 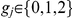 is the number of risk alleles at disease locus *j*, 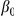 was chosen to lead to 5% of the population disease prevalence, and 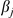
's were chosen to meet the specification for the six GRRs.

We simulated 15,000 replicate data sets. In each replication, genotypes of 314,174 whole-genome SNPs were generated for each of 500 unrelated cases and 500 unrelated controls. SNPs with Hardy-Weinberg exact *P* value<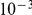 in the control group were excluded. *P* values were obtained using the one-degree-of-freedom chi-square test to compare the allele frequencies in cases and controls. The FDR level was to be controlled at 5%.

We followed Li et al. [11] to define the prioritized subsets according to the combinations of three factors: (Factor A) the number of prioritized regions (6, 14, 22); (Factor B) the size of each prioritized region (2 Mb, 20 Mb); (Factor C) the disease loci to be prioritized: (i) no disease loci; (ii) Locus 6; (iii) Loci 1 and 6; (iv) Loci 1, 2, 5, and 6; (v) Loci 4, 5, and 6; (vi) Loci 1, 4, 5, and 6; (vii) all except Locus 3; (viii) all loci. Factors A and B are directly related to the precision of a prioritization process, while Factor C is related to its accuracy.

## Results

### Simulation Results

#### FDR evaluations

**Figure 1:**
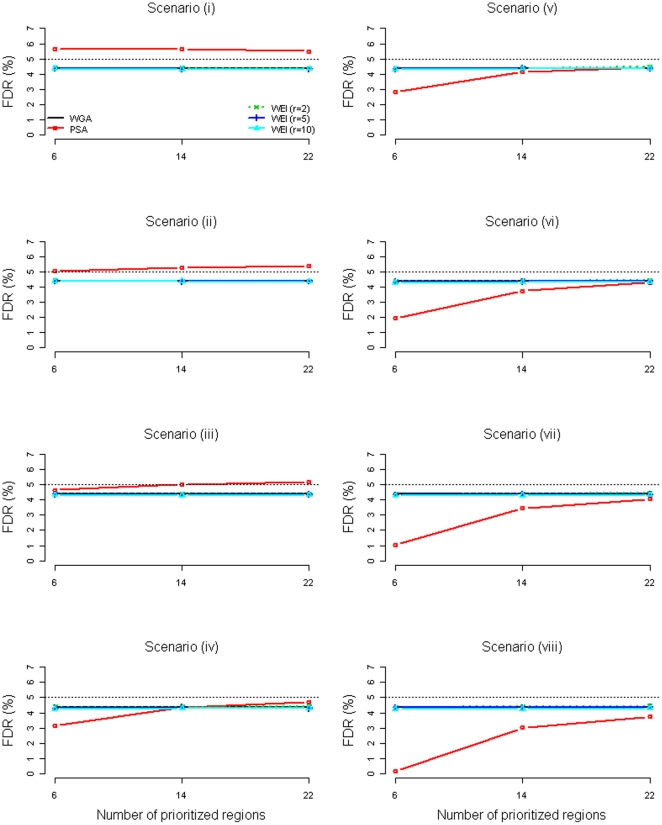
FDR of the WGA, the PSA, and the WEI (*r* = 2, 5, 10) when the prioritized region sizes were 2 Mb (without adjustment to the PSA).

**Figure 2:**
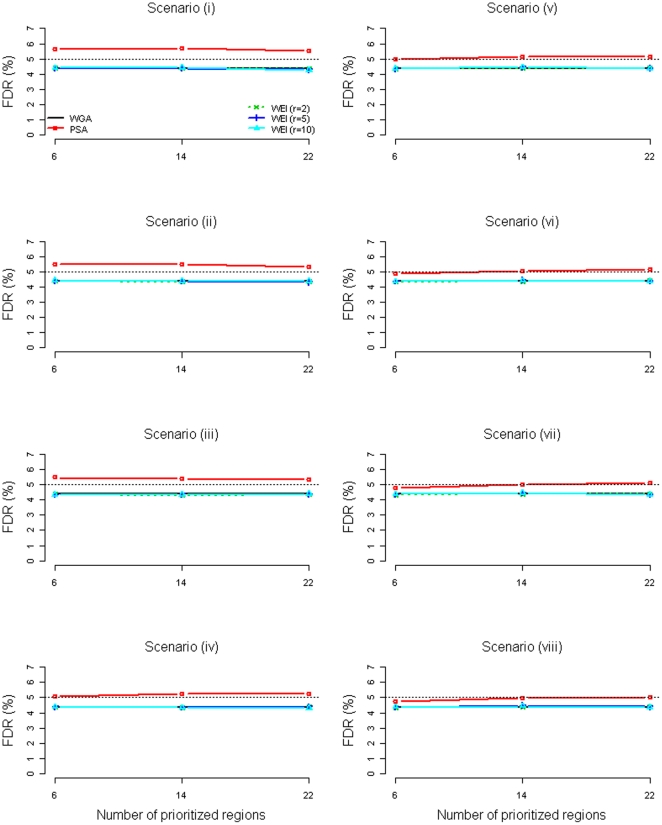
FDR of the WGA, the PSA, and the WEI (*r* = 2, 5, 10) when the prioritized region sizes were 20 Mb (without adjustment to the PSA).

Figures 1 and 2 present the FDR (the mean ratio of the number of false rejections to the number of total rejections, based on the 15,000 replications) of the WGA, the PSA, and the WEI (*r* = 2, 5, 10) when the prioritized region sizes were 2 Mb and 20 Mb, respectively. Note that the FDR of the PSA was the *overall* FDR, obtained by the mean ratio of the number of *total* false rejections *from the two subsets* to the number of *total* rejections *from the two subsets*. The WGA and the WEI had very similar FDRs, which were under control for all scenarios. For the PSA, accurate (true disease loci were prioritized) and precise (prioritized regions were narrower) prioritization led to lower *overall* FDR. Comparing the eight scenarios when six 2-Mb regions were prioritized (Figure 1), the more accurate the prioritization (more true disease loci were prioritized), the lower the *overall* FDR. However, within a same scenario (especially for Scenarios (iv)–(viii)), the *overall* FDRs were not as low when more regions unrelated to the disease were prioritized (14 or 22 regions compared to 6 regions). Comparing Figure 2 (where six 20-Mb regions were prioritized) with Figure 1 (where six 2-Mb regions were prioritized), the *overall* FDRs did not remain as low due to the decreasing precision of the prioritization (20 Mb compared to 2 Mb). The ability of the PSA to control the *overall* FDR depends on the accuracy and precision of the prioritization. When none of the true disease loci was prioritized (Scenario (i) of Figures 1 and 2), the *overall* FDR of the PSA was inflated to 5.6%. When only one or two disease loci were prioritized with higher precision (Scenarios (ii) & (iii) of Figure 1), the *overall* FDR of the PSA was ‘almost’ under control (still a slight inflation on the FDR). However, when the one or two disease loci were prioritized with lower precision (Scenarios (ii) & (iii) of Figure 2), the *overall* FDR of the PSA was inflated to 5.3%.

We see that the *overall* FDR of the PSA can be inflated to 5.6%. To have a fair evaluation on the power, we deliberately set the FDR control level at 4.4% (because the ratio of 5% to 5.6% is approximately equal to the ratio of 4.4% to 5%) for the PSA, under Scenarios (i)–(iii) for 2 Mb prioritized region size and under Scenarios (i)–(v) for 20 Mb size. After this adjustment, the *overall* FDRs of the PSA method were not larger than 5% (see our Supporting Information S1).

#### Power evaluations

**Figure 3:**
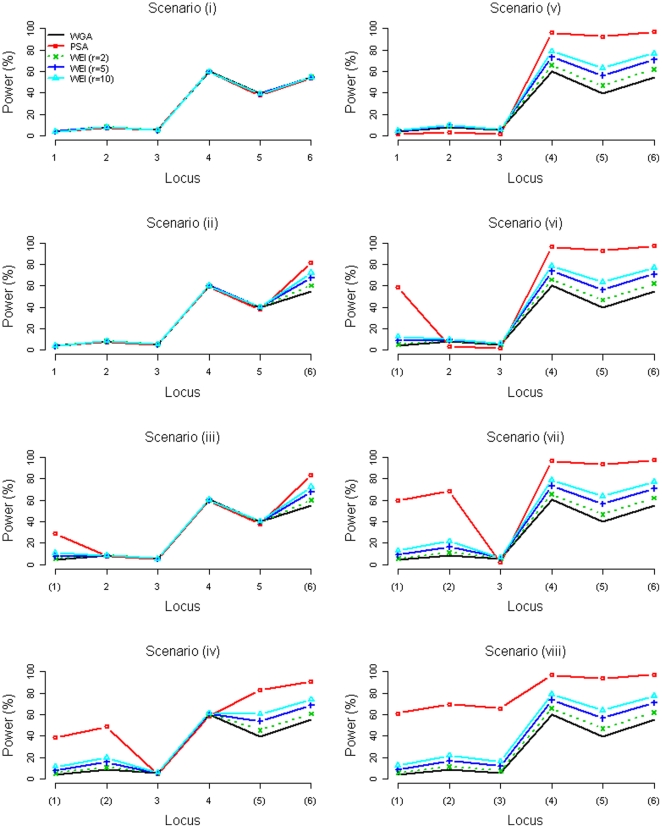
Power comparison between the WGA, the PSA, and the WEI (*r* = 2, 5, 10) when six 2-Mb regions were prioritized (with adjustment to the PSA). A locus with parentheses indicates that the disease locus was included in the prioritized subset.

**Figure 4:**
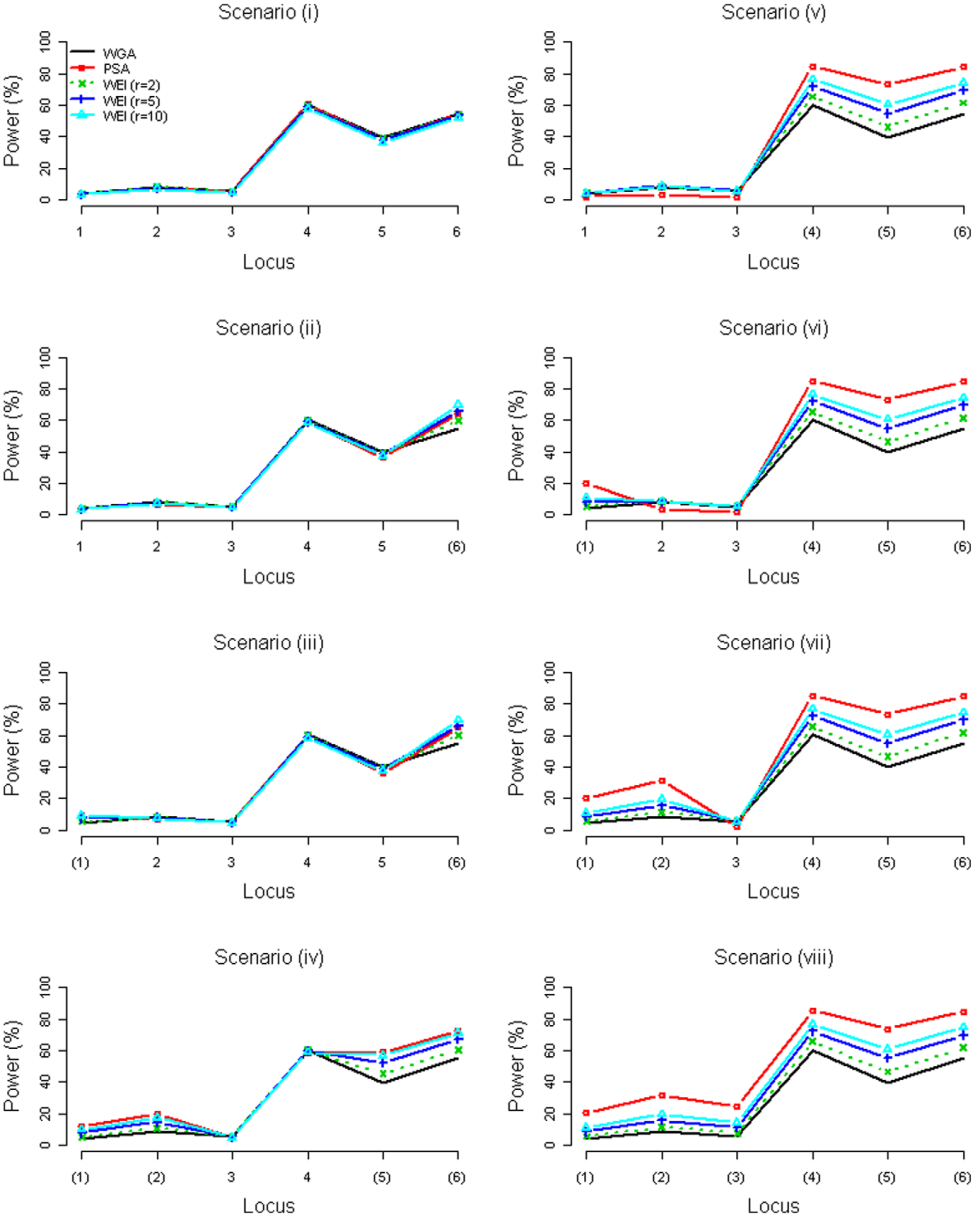
Power comparison between the WGA, the PSA, and the WEI (*r* = 2, 5, 10) when six 20-Mb regions were prioritized (with adjustment to the PSA). A locus with parentheses indicates that the disease locus was included in the prioritized subset.

Power of detecting a disease locus was defined as the proportion of ‘successful detections’ of that disease locus among all the 15,000 replications, in which a ‘successful detection’ was defined as ‘declaration of significance for at least one SNP within one Mb from the disease locus’ (following the definition by Li et al. [11]). Figures 3 and 4 present the power comparisons between the WGA, the PSA, and the WEI (*r* = 2, 5, 10) when six 2-Mb regions and when six 20-Mb regions were prioritized, respectively. Our Supporting Information S1 shows the results when 14 or 22 regions were prioritized, each region with a size of 2 Mb or 20 Mb. Note that the power was compared (Figures 3, 4, and Supporting Information S1) with the adjustment of FDR for the PSA (that is, the FDR control level was deliberately set at 4.4% for the PSA, under Scenarios (i)–(iii) for 2 Mb prioritized region size and under Scenarios (i)–(v) for 20 Mb size.).

The accuracy of a prioritization process directly affects the relative power performance of the three methods. When the regions encompassing the true disease loci were prioritized, the most powerful method was the PSA, then was the WEI (*r* = 10). The WEI with a larger *r* was more powerful than the WEI with a smaller *r* (see Scenarios (ii)–(viii) of Figures 3 & 4, where at least one disease locus was prioritized). When the regions encompassing the true disease loci were *not* prioritized, the power loss of the PSA and the WEI was almost negligible. The WEI with a larger *r* suffered from more power loss than the WEI with a smaller *r* (see Scenario (i) of the last figure in the Supporting Information S1, where no disease loci were prioritized).

Furthermore, the precision of a prioritization process also influences the power of the PSA and the WEI. Regarding the size of each prioritized region (2 Mb or 20 Mb) and the number of prioritized regions (6, 14, or 22), the power improvement of the PSA and the WEI was not as prominent when the prioritization was not as precise, i.e., a wider region around a true disease locus was prioritized (20 Mb compared to 2 Mb), or more regions unrelated to the disease were prioritized (22 or 14 regions compared to 6 regions).

### Application to the Wellcome Trust Case Control Consortium (WTCCC) Data

We further demonstrate the WGA, the PSA, and the WEI with a GWAS on type 1 diabetes (T1D). The data set was provided by the Wellcome Trust Case Control Consortium (WTCCC) [17] that included 2,000 T1D cases and 3,000 controls. Subjects were living within England, Scotland, and Wales (‘Great Britain’). The vast majority had self-identified themselves as white Europeans [17]. The control subjects were from 1958 British Birth Cohort (1,500 subjects) and UK Blood Services sample (1,500 subjects). After excluding subjects identified as having recent non-European ancestry, there were 1,963 T1D cases and 2,938 controls [17]. Subjects were genotyped using the Affymetrix GeneChip 500 K arrays comprising 500,568 SNPs. According to the WTCCC criteria [17], 459,653 SNPs passed the quality control filters. We further removed 578 SNPs with poor clustering and retained 459,075 SNPs.

#### WGA

**Figure 5:**
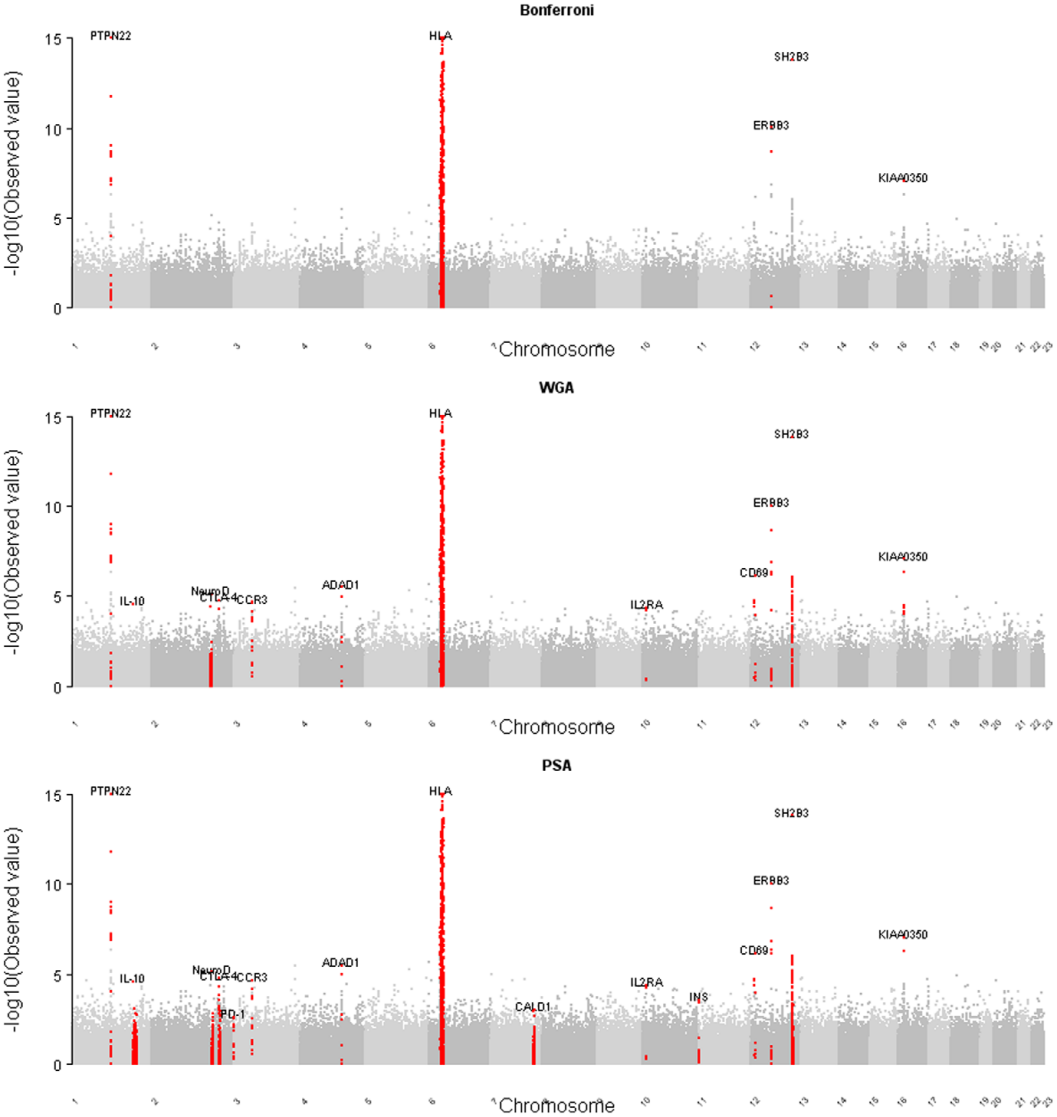
Manhattan plot for the T1D data set. The *x*-axis lists the chromosome numbers, and the *y*-axis presents 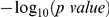. The red points label the significant genes identified by each method (from top to bottom: the Bonferroni correction to control the FWER at 5%, the WGA to control the FDR at 5%, and the PSA to control the FDR at 5%). This figure was plotted with the R package ‘gap’ [31].

For each SNP, we used the *p* value obtained from the genotypic test. Controlling the FDR at 5%, 12 independent association signals were declared to be significant with the WGA (an association signal was identified given more than a single significant SNP within 2 Mb). Figure 5 and Table 1 show the 12 signals that can be mapped to 12 genes. Among them, five were declared to be significant when the Bonferroni correction was used to control the FWER at 5%.

**Table 1:** Results of the T1D data set.

Gene^1^	Chromosome	Bonferroni^2^	WGA,WEI (*r* = 2)	WEI (*r* = 5, 10)	PSA	WTCCC SNP^3^	Genotypic *P* value^4^	Supported by later studies^5^
HLA*	6p21	V	V	V	V	rs9272346	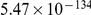	[32]–[34]
PTPN22*	1p13	V	V	V	V	rs6679677	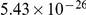	[22], [35]–[49]
SH2B3	12q24	V	V	V	V	rs17696736	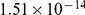	[50]
ERBB3	12q13	V	V	V	V	rs11171739	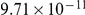	[51]
KIAA0350	16p13	V	V	V	V	rs12708716	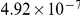	[52], [53]
CD69	12p13		V	V	V	rs11052552	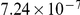	[54]
ADAD1	4q27		V	V	V	rs17388568	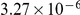	[55]–[57]
NeuroD*	2q32		V	V	V	rs10206282	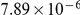	[58], [59]
CTLA4*	2q33		V	V	V	rs231726	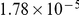	[19], [60]–[63]
CCR3	3p21		V	V	V	rs2157057	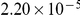	[64]
IL-10*	1q31–q32		V	V	V	rs12061474	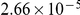	[65]–[70]
IL2RA*	10p15–p14		V	V	V	rs2104286	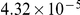	[71]–[76]
INS*	11p15			V	V	rs6578252	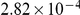	[19]–[22]
CALD1*	7q33				V	rs2250603	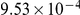	[23], [24]
PD-1*	2q37				V	rs10192057	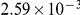	[25], [26]
Number of significant genes		5	12	13	15			

1 * : being allocated in the prioritized subset.

2 V: significant.

3 The WTCCC SNP showing the strongest association evidence in that region.

4 The *p* value of the genotypic test of the WTCCC SNP showing the strongest association evidence in that region.

5 That association signal is supported by later studies that have *NOT* been selected as our prior information (in Table 2).

#### Prioritization process

To perform the PSA and the WEI, prior information is required to assign each SNP to be ‘more likely a true positive’ or ‘more likely a true negative’. We collected the information by searching for publications with ‘gene’, ‘association’, and ‘type 1 diabetes’ in their titles. PubMed shows 89 publications meeting this searching criterion. Among them, we used the studies independent of the WTCCC project and published *prior to* the WTCCC publication [17]. Table 2 lists the genes that are previously reported to be associated with T1D. We obtained the physical position of each gene (listed in Table 2) from the Gene Location website (http://genecards.weizmann.ac.il/geneloc/index.shtml). SNPs within 1 Mb from each gene were prioritized (so the size of each prioritized region was 2 Mb). When prioritizing SNPs according to prior information, there is no stringent criterion for the sizes of prioritized regions (we will discuss this in the Discussion). However, in general, a wider region should be prioritized for a linkage peak than that for an association signal, because linkage is a coarse mapping while association is a fine mapping. In this data analysis, our prior information came from previous association studies on T1D, and therefore we chose a moderate prioritization size for each region −2 Mb. Totally, we prioritized 6,914 SNPs and left the remaining 452,161 SNPs in the non-prioritized subset. A list of the prioritized SNPs is available upon request.

**Table 2:** Prior knowledge: genes that are previously reported to be associated with type 1 diabetes.

Gene	Chromosome	Start base pair^1^	End base pair^1^	Publications prior to the WTCCC paper
PTPN22	1	114356433	114414381	[77]–[79]
IL-10	1	206940947	206945839	[80]
IL1R1	2	102681004	102744178	[81]
NeuroD	2	182537815	182545603	[82]
CD28	2	204571198	204738683	[83]
CTLA4	2	204732509	204738683	[83]–[90]
SLC11A1^2^	2	219246752	219261617	[91], [92]
PD-1	2	242792033	242801060	[93]
DBP	4	72607410	72669758	[94]
MIC-A	6	31367561	31433586	[95]
HLA-DRB1	6	32546546	32557625	[96], [97]
HLA-DQA1	6	32595956	32714992	[97]
HLA-DQB1	6	32627244	32731330	[97]
SUMO4	6	149721495	149722182	[98]
MTH1	7	2281857	2291004	[99]
CALD1	7	134429003	134655480	[100]
IL2RA	10	6052652	6104333	[101]
INS	11	2181009	2182571	[102], [103]
IL-18	11	112013974	112034840	[85], [104]
VDR	12	48235320	48336831	[105]–[107]
OAS	12	113344582	113369991	[108]
HSD11B2	16	67465036	67471456	[109]
ICAM-1	19	10381517	10397291	[110], [111]

1 The physical positions were obtained from the Gene Location website (http://genecards.weizmann.ac.il/geneloc/index.shtml).

2 Other aliases: LSH, NRAMP, NRAMP1.

#### PSA

To perform a PSA, the genotypic *p* values of the 459,075 SNPs were accordingly allocated into two subsets. The FDR was to be controlled at 5%. The Benjamini and Hochberg's FDR controlling [2] was applied to the two subsets of *p* values, respectively. Finally, 15 genes were declared to be significant (see Figure 5 and Table 1). Among them, nine genes came from the prioritized subset, including *HLA*, *PTPN22*, *NeuroD*, *CTLA4*, *IL-10*, *IL2RA*, *INS*, *CALD1*, and *PD-1*. The last three were not identified as significant genes with the WGA method. Being allocated into the prioritized subset, they (*INS*, *CALD1*, and *PD-1*) had a chance to be identified as significant genes. We further evaluated whether the FDR was well controlled in the prioritized subset. We permuted the phenotypes 10^4^ times and obtained 10^4^ null *p* values for each of the 6,914 SNPs. Then we estimated the number of false positives by counting the number of null *p* values more extreme (i.e., smaller) than 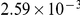, the largest *P* value of the association signals in the prioritized subset. In this way, we obtained an estimated permutation-based FDR [18] at 3.64%, still less than the FDR control level of 5%. In fact, several studies published later than the WTCCC paper [17] also supported the association of these three genes with T1D (*INS* [19]–[22], *CALD1* [23], [24], and *PD-1* [25], [26]).

#### WEI

To perform a WEI, in addition to the prior information used for the PSA, a ratio of the two weights (*r*) was specified at 2, 5, and 10, respectively. In this T1D study, the proportion of SNPs thought to be more likely true positives among all the SNPs was 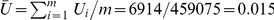. With a specified *r*, the weight given to SNPs thought to be more likely true positives was 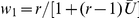, and that given to SNPs thought to be more likely true negatives was 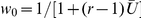. The *p* values were then weighted according to 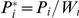, where 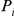 and 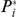 were respectively the original and weighted *p* values of the *i*th SNP, and 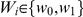. We used the Benjamini and Hochberg's method [2] to control the FDR at 5%. When *r* was specified at 2, the WEI identified the same 12 genes as the WGA. When *r* was specified at 5 or 10, the WEI identified one more gene – *INS* (see Table 1).

## Discussion

The PSA and the WEI both require prior knowledge to boost the power of detecting signals. Prior knowledge can be collected from previous independent studies that were not based on the same data of the current working study. It should be searched *before* seeing the analysis results of the individual tests in the current study. Both the PSA and the WEI have caught much attention in the era of high-throughput genomics, for their improvement on the FDR control. With the advancement of biological technologies, we now can obtain substantial genomic data with decreasing costs. To facilitate discoveries from more and more hypothesis tests, clarifying the merits and limitations of the PSA and the WEI is important.

The PSA and the WEI both can increase power when the prior is informative. As shown in our simulation, the PSA can especially create substantial power improvements given accurate and precise prioritization. When researchers fail to prioritize some true disease loci, the power loss of the PSA and the WEI is almost negligible. Like the WGA, the WEI has a solid theoretical background [10] and a good ability to control the FDR at the desired level (Figures 1 and 2).

Although the PSA can increase much power when the prior is informative, its *overall* FDR can be slightly inflated if the prioritization is not accurate and precise. In our simulation, the *overall* FDR of the PSA was obtained by the mean ratio of the number of *total* false rejections *from the two subsets* to the number of *total* rejections *from the two subsets*, based on 15,000 replications. Because the Benjamini and Hochberg's FDR controlling [2] is applied to the prioritized and the non-prioritized subsets *respectively*, the *overall* FDR of the PSA is not guaranteed to be controlled at the desired level even when the numbers of SNPs in the two subsets are both large enough. For example, when 22 20-Mb regions (not precise) were prioritized, the *overall* FDRs of the PSA were inflated under Scenarios (i)–(iv) (not accurate) despite ∼49,000 SNPs in the prioritized subset and ∼265,000 SNPs in the non-prioritized subset. This is because when the prioritization is not accurate and precise, the number of true positives from the prioritized subset can be small (or the number of false positives can be large). This can inflate the *overall* FDR of the PSA.

There is no stringent criterion for the sizes of prioritized regions, when prioritizing SNPs according to prior information. Given a linkage peak or an association signal based on prior studies, prioritizing a narrow region may miss the true disease loci and lose the accuracy, while prioritizing a wide region may lose the precision. It is not easy to conclude how wide a region should be prioritized. Although the determination for a region size is somewhat *ad hoc*, a general principle is to estimate the permutation-based FDR [18] after performing the PSA. This can empirically evaluate whether the FDR within each subset is well controlled.

The WEI can be equipped with ‘general weighting scheme’, although we only evaluated the ‘binary weighting scheme’ for its parallel comparison with the PSA. For the general weighting scheme, each test is assigned a specific weight, not only either 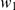 or 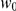. In this way, the WEI is more flexible than the PSA in the sense that the weights can be assigned in a continuous scale. To mimic this flexibility, the PSA can extend its original concept to allocate SNPs into more than two subsets. However, this will inevitably increase the possibility of unsatisfactory FDR control.

In addition to conventional GWAS, weighting tests can provide insights to rare variant detection. In the past several years, GWAS have identified hundreds of common genetic variants (minor allele frequency (MAF)>5%) for complex human diseases [27]. However, these common variants can only explain a small proportion of heritability. The field of genetic epidemiology is shifting toward the study of low-frequency (MAF 1%–5%) and rare variants (MAF<1%), which are thought to have larger effect sizes than common variants [28]. Unfortunately, rare variants are difficult to detect due to their low frequencies. Recently, a weighted-Holm procedure was shown to substantially improve the power of detecting rare variants with large genetic effects [29]. Furthermore, a study has shown that low-frequency variants can be identified by up-weighting SNPs with lower MAFs and then performing the FDR control [30]. Appropriately weighting genetic variants according to their MAFs can facilitate the detection of rare variants. Applying the PSA and the WEI to this topic deserves further investigation.

## Supporting Information

Supporting Information S1 **FDR of the WGA, the PSA, and the WEI (** ***r*** **= 2, 5, 10) when the prioritized region sizes were 2 Mb and 20 Mb (with adjustment to the PSA), respectively; power comparison between the WGA, the PSA, and the WEI (** ***r*** **= 2, 5, 10) when 14 2-Mb, 14 20-Mb, 22 2-Mb, and 22 20-Mb regions were prioritized (with adjustment to the PSA), respectively.**
(DOC)
