# Supplementary material for: Improving Power of Genome-Wide Association Studies with Weighted False Discovery Rate Control and Prioritized Subset Analysis
Source: PLoS One. 2012 Apr 9;7(4):e33716. doi: 10.1371/journal.pone.0033716 (PMC3322139; doi:10.1371/journal.pone.0033716)
Supplement: Supporting Information S1 — FDR of the WGA, the PSA, and the WEI ( r = 2, 5, 10) when the prioritized region sizes were 2 Mb and 20 Mb (with adjustment to the PSA), respectively; power comparison between the WGA, the PSA, and the WEI ( r = 2, 5, 10) when 14 2-Mb, 14 20-Mb, 22 2-Mb, and 22 20-Mb regions were prioritized (with adjustment to the PSA), respectively. (DOC) [file pone.0033716.s001.doc]

**Supporting Information S1 of**

**Improving Power of Genome-wide Association Studies with Weighted False Discovery Rate Control and Prioritized Subset Analysis**

### Wan-Yu Lin ^1,2 §^, Wen-Chung Lee ^1,3^

^1^ Institute of Epidemiology and Preventive Medicine, College of Public Health, National Taiwan University, No. 17, Xuzhou Rd., Taipei 100, Taiwan.

^2^ Department of Biostatistics, University of Alabama at Birmingham, 1665 University Boulevard, Birmingham, Alabama 35294, U.S.A.

^3^ Research Center for Genes, Environment and Human Health, National Taiwan University, No. 17, Xuzhou Rd., Taipei 100, Taiwan.

^§^Corresponding author:

# Wan-Yu Lin, Ph.D.

# RPHB 327, 1530 3^rd^ Ave S, Birmingham, AL 35294-0022

# Phone: (205) 975-9207 Fax: (205) 975-2540

E-mail: wlin@uab.edu


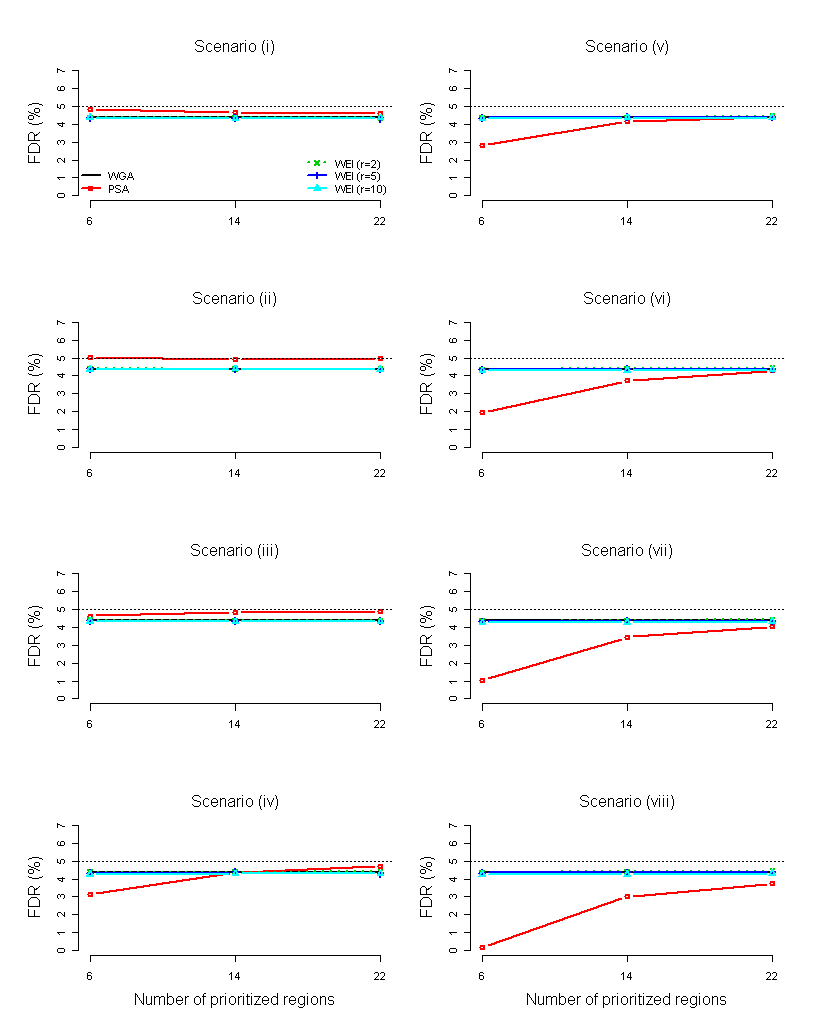


FDR of the WGA, the PSA, and the WEI (*r* = 2, 5, 10) when the prioritized region sizes were 2 Mb (with adjustment to the PSA)


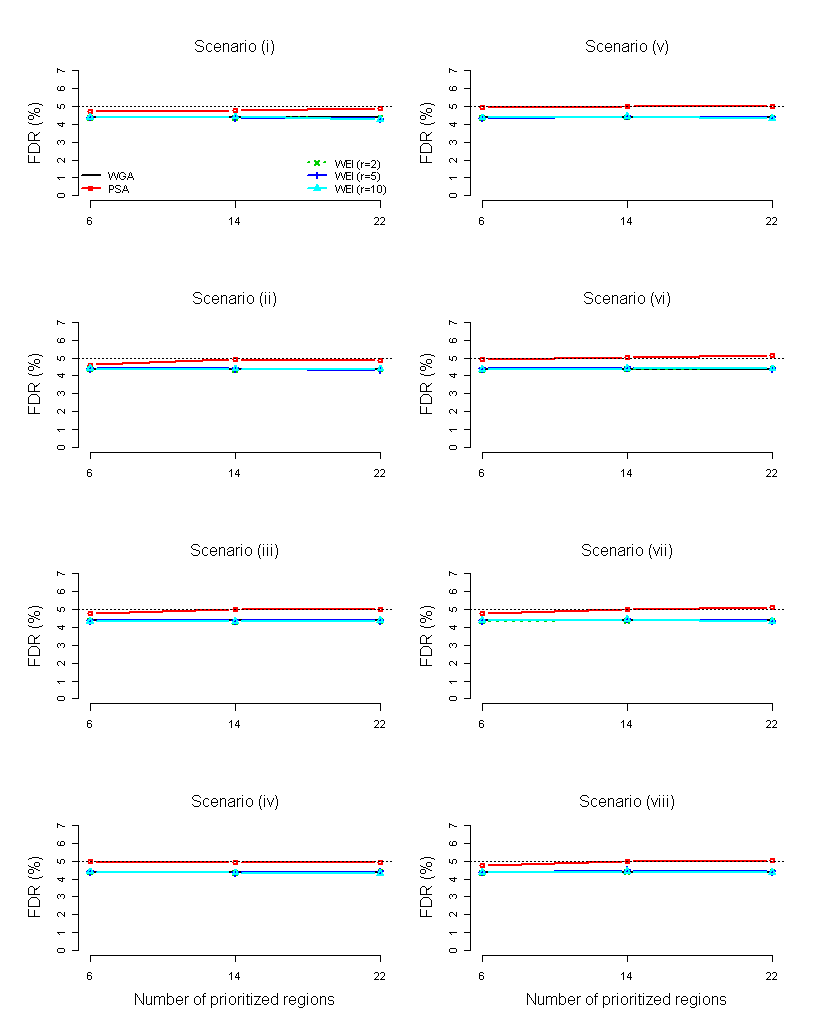


FDR of the WGA, the PSA, and the WEI (*r* = 2, 5, 10) when the prioritized region sizes were 20 Mb (with adjustment to the PSA)


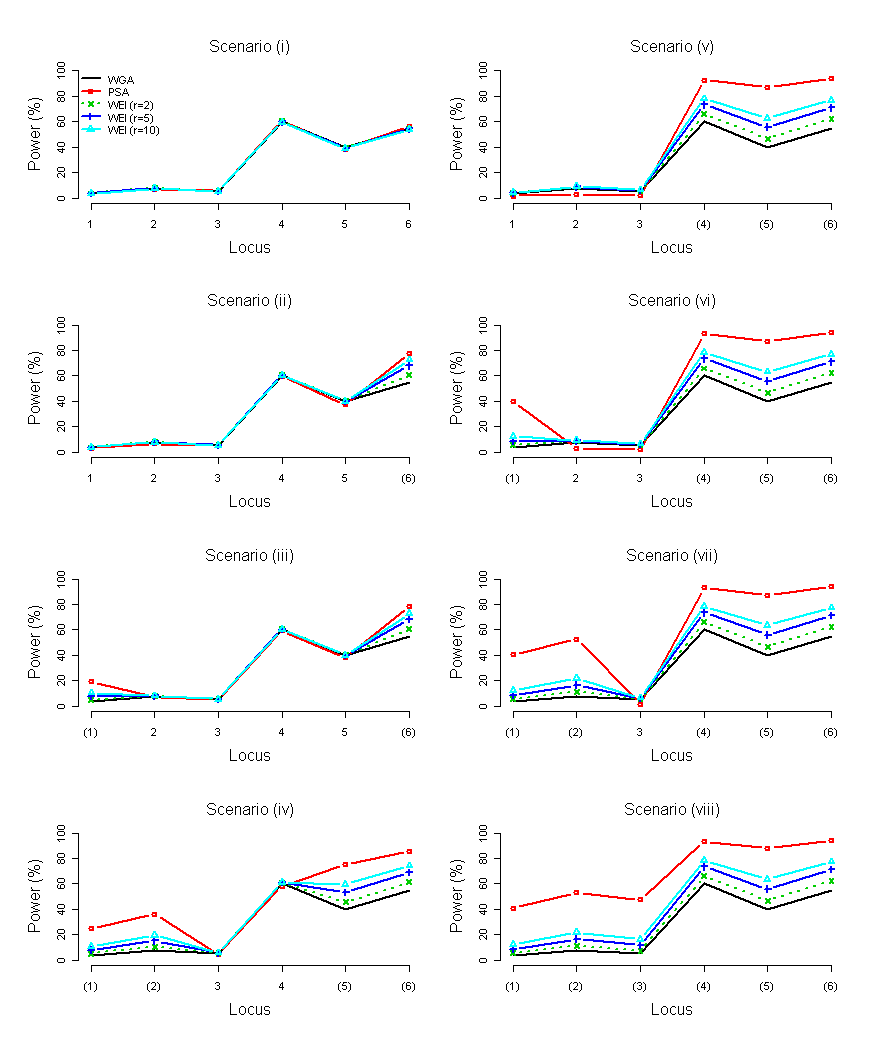


## Power comparison between the WGA, the PSA, and the WEI (r = 2, 5, 10) when 14 2-Mb regions were prioritized (with adjustment to the PSA) (A locus with parentheses indicates that the disease locus was included in the prioritized subset.)


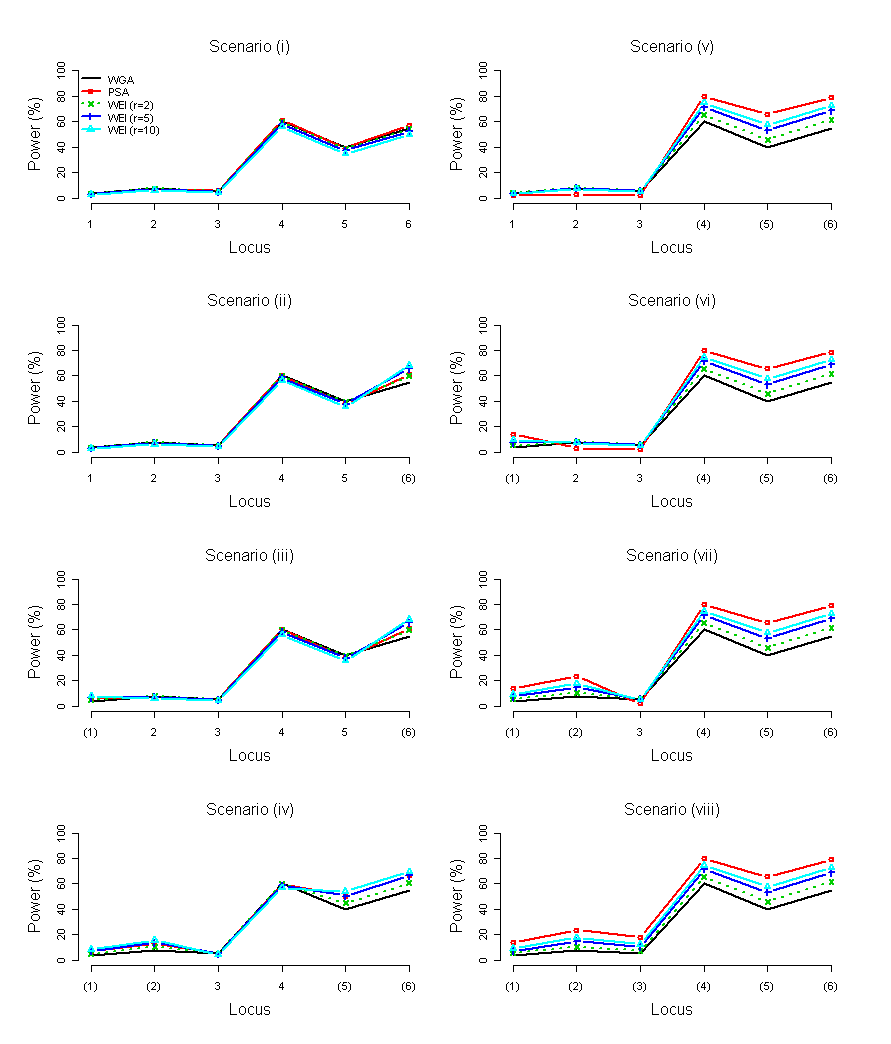


Power comparison between the WGA, the PSA, and the WEI (*r* = 2, 5, 10) when 14 20-Mb regions were prioritized (with adjustment to the PSA) (A locus with parentheses indicates that the disease locus was included in the prioritized subset.)


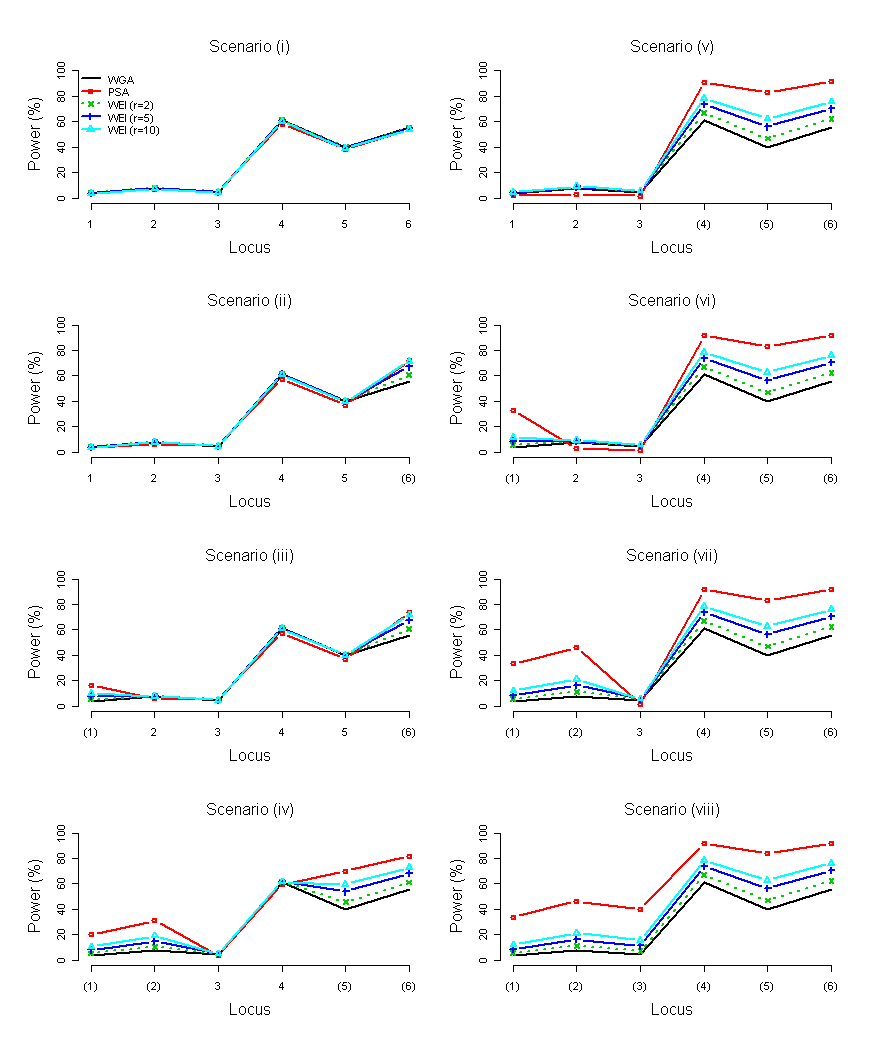


Power comparison between the WGA, the PSA, and the WEI (*r* = 2, 5, 10) when 22 2-Mb regions were prioritized (with adjustment to the PSA) (A locus with parentheses indicates that the disease locus was included in the prioritized subset.)


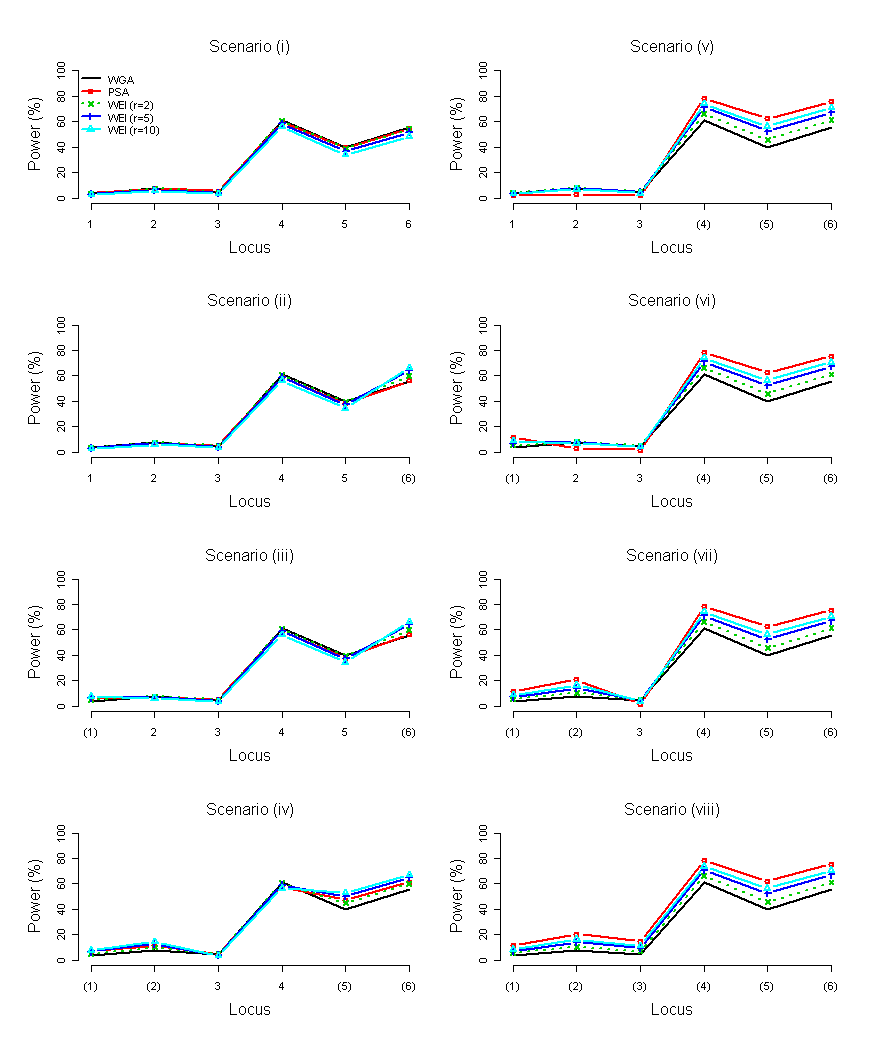


Power comparison between the WGA, the PSA, and the WEI (*r* = 2, 5, 10) when 22 20-Mb regions were prioritized (with adjustment to the PSA) (A locus with parentheses indicates that the disease locus was included in the prioritized subset.)
